# Supplementary material for: Dynamics of self-reorganization explains passivation of silicate glasses
Source: Nat Commun. 2018 Jun 4;9:2169. doi: 10.1038/s41467-018-04511-2 (PMC5986767; doi:10.1038/s41467-018-04511-2)
Supplement: Supplementary file 1 — Supplementary Information [file 41467_2018_4511_MOESM1_ESM.pdf]

Supplementary Information

Dynamics of self-reorganization explains passivation of silicate glasses

Stéphane Gin[1,‡], Marie Collin[1], Patrick Jollivet[1], Maxime Fournier[1], Yves Minet[1], Laurent Dupuy[2], Thiruvilla Mahadevan[3], Sebastien Kerisit[4], Jincheng Du[3]

[1] CEA, DEN, DE2D, SEVT, F-30207 Bagnols sur Cèze, France

[2] Tescan Analytics, ZAC St Charles, 13710 Fuveau, France

[3] Department of Materials Science and Engineering, University of North Texas, Denton, TX 76203, USA

[4] Pacific Northwest National Laboratory, Physical & Computational Sciences Directorate, Richland, WA 99352, USA

[‡] e-mail: stephane.gin@cea.fr

Supplementary Note 1: Water quantification and speciation analysis

Sample preparation and analytical techniques

All experiments were performed on an ISG batch prepared by MoSci Corporation (Rolla MO, USA). The glass was crushed, and the powder was sieved and washed in acetone and absolute ethanol to remove fine particles. Leaching experiment was conducted on the 3.5 – 5.5 µm size fraction to obtain a totally altered glass (called 'gel' hereafter) powder for solid characterization.

Glass leaching was performed in a perfluoroalkoxy (PFA) vessel using 18.2 MΩ·cm deionized water initially saturated with respect to amorphous silica (C_0(Si) = 143 mg·L^{-1}, pH$_{90\,°C}$ 7) and containing K = 0.5 g·L^{-1} introduced as potassium chloride. SiO_2 was introduced under agitation at 90 °C until total dissolution. During the experiment, pH was maintained at 7.0 ± 0.5 by adding small quantities of a 0.5 N nitric acid solution.

A small fraction of completely altered 3.5 – 5.5 µm glass powder was dissolved by an acid solution (10 HCl + 5 HNO_3 + 4 HF). The resulting solution was analyzed by ICP-OES (Thermo Scientific iCAP™ 6000 Series) to quantify Si, B, Na, Al, Ca, Zr, and K in the altered glass. Two analyses were conducted for repeatability. Oxygen in the altered glass was considered using the following equation:

$$\mathrm{O}_{\,altered\ glass} = \sum \mathrm{O}_{i\ pristine\ glass} \cdot \frac{[i]_{\,altered\ glass}}{[i]_{\,pristine\ glass}} \quad (1)$$

where i = Si, B, Na, Al, Ca, or Zr and $\mathrm{O}_{i\ pristine\ glass}$ is the number of oxygen atoms associated with each element i in the pristine glass.

Thermogravimetric analyses (TGA) were performed on a Seratum TG-DSC 92-16.18, under a flow of argon in aluminum oxide crucible. Both 3.5 – 5.5 µm pristine glass and gel powders were analyzed

(~ 70 mg for each). The gel was dried at 50 °C for 24 hours prior TGA. For pristine glass, a classical heating ramp at 10 °C·min^{-1} was used between room temperature and 1200 °C. For the gel, a faster heating ramp (30 °C·min^{-1}) was used to attain specific temperatures (90, 150, 300 and 450 °C), followed for each of these temperatures by a two-hour plateau. A last heating ramp at 10 °C·min^{-1} was then performed between 450 °C and 1200 °C.

^{1}H magic-angle spinning (MAS) nuclear magnetic resonance (NMR) analyses were performed on a Bruker Avance WB 300 MHz spectrometer (magnetic field 7.05 T). A Bruker 4 mm (external diameter of the ZrO_2 rotor) cross polarization magic-angle spinning (CP-MAS) probe was used at a sample rotation frequency of 10 kHz. The average sample mass was 90 mg. ^{1}H MAS NMR signal were obtained on gel by direct acquisition (DA), and using a Hahn Echo sequence (HE) with varying rotor-synchronized echo delays ranging from 100 to 80000 µs. ^{1}H NMR signal deconvolution was completed using the Dmfit program, considering a mixed Gaussian/Lorenzian model. Using HE sequence data, each contribution's position was fixed, and the width and amplitude were left free. The automatic optimization was run until reaching convergence.

Water content

Results of TGA are displayed in Supplementary Table 1 and Supplementary Figure 1 shows ^{1}H NMR analysis of the gel.

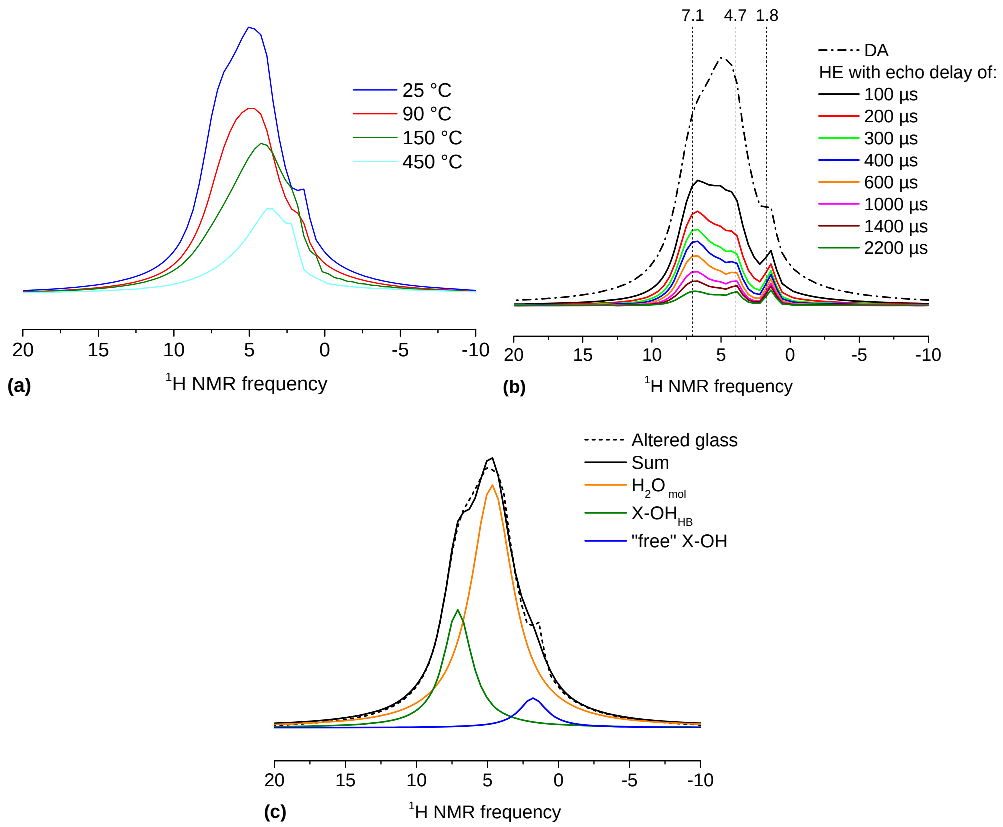

Supplementary Figure 1 – NMR analysis of the gel. **a** ^{1}H MAS NMR spectra of the 3.5 – 5.5 fully altered glass powder were acquired before and after heat treatments. Spectra are normalized to the sample mass. This series is used to estimate the number of contributions that need to be introduced for signal deconvolution. **b** ^{1}H MAS NMR signal obtained by direct acquisition (DA) and using a Hahn Echo pulse sequence (HE) with varying echo delays at ambient temperature. Using this sequence, the position of the contributions can be fixed. **c** ^{1}H NMR signal (recorded at 25 °C) deconvoluted using three contributions: one for hydrogen from water molecules H_2O_{mol}, one for hydrogen from H-bonded hydroxyls groups *X*-OH_{HB}, and one for hydrogen from non H-bonded hydroxyls groups "free" *X*-OH.

Supplementary Table 1 – TGA data.

	Pristine Glass	Gel
**Total water content (wt%)**	1.2 ± 0.1	12.5 ± 0.1

The deconvolution of the ^{1}H MAS NMR spectrum of the gel with 3 contributions gives the following species repartition (Table S2):

Supplementary Table 2 – Water speciation in altered glass (%). *X* refers to Si, Al, or Zr.

H of –*X*-OH$_{HB}$	H of H$_2$O$_{mol}$	H of "free" –*X*-OH
23.5	70.7	5.8

Volume, surface of the porosity and mean pore size

TGA data gives a direct access to the total quantity of proton per mass unit of sample, $n_{\mathrm{H}_{tot}}$:

$$n_{\mathrm{H}_{tot}} = 2\frac{\Delta_{\mathrm{H_2O}\,tot}}{M_{\mathrm{H_2O}}} \tag{2}$$

where $\Delta_{\mathrm{H_2O}\,tot}$ is the total mass of water lost in the gel measured by TGA and $M_{\mathrm{H_2O}}$ the water molar mass. Based on the proton species repartition obtained by NMR, molecular water and hydroxyl species n_{OH} per mass unit of gel can be quantified:

$$n_{\mathrm{H_2O}} = \frac{1}{2}\cdot n_{\mathrm{H}_{\mathrm{H_2O}}} = x\cdot n_{\mathrm{H}_{tot}} \tag{3}$$

where *x* is percentage of proton from H_2O_{mol}.

$$n_{\mathrm{OH}} = n_{\mathrm{H}_{\mathrm{OH}}} = y\cdot n_{\mathrm{H}_{tot}} \tag{4}$$

where *y* is the percentage of protons from *X*-OH$_{HB}$ and “free” *X*-OH combined. Assuming that the effective pore volume corresponds to the volume given by water molecules only ($V_{p_{effective}}$), while the total volume ($V_{p_{total}}$) is a combination of water and hydroxyl volume:

$$V_{p_{effective}} \approx \frac{n_{\mathrm{H_2O}}.M_{\mathrm{H_2O}}}{\rho_{\mathrm{H_2O}}} \tag{5}$$

$$V_{p_{total}} \approx \frac{n_{\mathrm{H_2O}}.M_{\mathrm{H_2O}}}{\rho_{\mathrm{H_2O}}} + \frac{n_{\mathrm{OH}}.M_{\mathrm{OH}}}{\rho_{\mathrm{OH}}} \tag{6}$$

where $\rho_{\mathrm{H_2O}}$ is the density of water, M_{OH} the molar mass of hydroxyl, and ρ_{OH} the density of hydroxyl groups. The literature indicates that a fully hydroxylated surface of amorphous silica has a density of ~ 5 OH groups per square nm[1,2]. Assuming our gel has the same density of terminal OH, the specific surface of the gel (S_p) is given by:

$$S_p = n_{\mathrm{OH}}\cdot N_A\cdot\frac{1}{5} \tag{7}$$

where N_A is the Avogadro number. It is worth noting that this estimation combines open and closed porosity. Assuming an unimodal distribution of spherical pores, both specific surface (S_p) and specific volume (V_p) of the porosity can be defined. Combining them, it is possible to calculate the mean pore diameter:

$$S_p = n \cdot \pi d^2 \quad (8)$$

$$V_p = n \cdot \frac{4\pi}{3} \cdot \left(\frac{d}{2}\right)^3 \quad (9)$$

$$d = 6 \cdot \frac{V_p}{S_p} \quad (10)$$

where *n* is the number of pores and *d* the mean pore diameter. The obtained data are displayed in Supplementary Table 3:

Supplementary Table 3 – Surface area and porosity characteristic of the gel.

Parameters of the gel porosity				
		**Specific surface ($m^2 \cdot g^{-1}$)**	**"Free" volume (%)**	**Mean pore diameter (nm)**
**Mobile elements release**		-	32 ± 6	-
**TGA/NMR**	**Effective** (H_2O)	489 ± 98	22 ± 4	1.1
	**Total** (H_2O + OH)		40 ± 7	2.0

Oxygen repartition in the gel

Combining all the data obtained from chemical analysis, TGA and NMR, it is possible to calculate the oxygen distribution in the gel considering 3 species:

- $n_{\mathrm{O\ BO}}$: bridging oxygen BO, also called $O_{network}$ (skeleton of the gel),
- $n_{\mathrm{O\ NBO}}$: non-bridging oxygen NBO (hydroxyl),
- $n_{\mathrm{O\ H_2O}}$: oxygen from water molecule.

O of water molecule, $n_{\mathrm{O\ H_2O}}$ is simply $n_{\mathrm{H_2O}}$ given by equation 3, and O of terminal OH groups, $n_{\mathrm{O\ NBO}}$ corresponds to n_{OH} given by equation 4. The chemical analysis of the gel (Table 1) gives by calculation $n_{\mathrm{O\ NBO}} + n_{\mathrm{O\ BO}}$. This third entry data enables the calculation of $n_{\mathrm{O\ BO}}$. Supplementary Table 4 displays the calculated values of the 3 types of O species in the gel.

Supplementary Table 4 – O distribution in the gel (%).

**$O_{network}$**	**O of H_2O_{mol}**	**O of –*X*-OH**
71.6	12.9	15.5

Supplementary Note 2 – Validation of ToF-SIMS depth profiling analyses

In order to validate the $^{18}O/^{16}O$ profiles presented in this work, two complementary studies were performed. The first one attempted to minimize the pore water mobility during sample preparation and analysis by freezing the sample immediately after the tracing experiment and keeping the sample frozen during ToF-SIMS depth profiling analysis. The second one aimed at verifying the symmetry of the $^{18}O/^{16}O$ profile (among other profiles) when the altered layer was sputtered in the two opposite directions (from the solution toward the pristine glass on the one hand, and from the pristine glass toward the solution on the other hand), to verify the absence of artefact in the quantification of the isotopic profiles. For this second study, a thin flake of ISG glass was altered and the two faces were analyzed in one measurement.

Cryo-ToF-SIMS analysis

A chunk of the sample ISG-363 (ISG glass sample altered for 363 days at 90 °C in a solution saturated with amorphous silica and at pH 7) was immersed for 3 hours at room temperature in a solution composed of 80 % $H_2{}^{18}O$ 20 % D_2O. This tracing experiment was conducted under a dry and inert atmosphere in a glove box. After 3 hours, the sample was taken out of the tagged solution, blow dried to remove water adsorbed on the external surfaces, plunged in liquid N_2 (77 K) for 30 minutes, and then mounted on a cooled SIMS sample holder. Finally, the holder was placed in the vacuum chamber. This operation let the frozen sample in contact with air for approximately 1 minute.

ToF-SIMS analyses were obtained using a ToF-SIMS5 spectrometer (IonTof – Münster, Germany). The spectrometer was operated at a pressure of 6 10^{-8} mbar. A pulsed 25 keV ~ 0.1 pA $Bi_3{}^{++}$ primary ion source was employed for $^{18}O/^{16}O$ analysis and a pulsed 25 keV ~ 4 pA Bi^+ for D/H analysis over a rastered area of 50 µm × 50 µm (beam size ≤ 3 µm). Depth profiling was performed using a 2 keV Cs^+ sputter beam with a 45° incidence to the specimen surface giving a 195 nA target current over a 200 µm x 200 µm area. A flood gun was used to compensate the charging effect on the surface. Negative ion depth profiles were recorded. Data acquisition and post-processing analyses were performed using the SurfaceLab 6 software. A profilometer was used to measure the crater depth at the end of the analysis. Data are displayed as a function of depth considering the same sputtering rate in the alteration layer and pristine glass.

Supplementary Figure 2 shows that both $H_2{}^{18}O$ and D_2O have diffused though the alteration layer up to the reaction front, well-marked by the sharp B drop. The mean values of $^{18}O/^{16}O$ and D/H in the alteration layer are 0.012 and 0.013, respectively. These values remain far from those in the tracing solution, but high relative to the natural abundance expected before the tracing experiment ($2\cdot10^{-3}$ and $1.1\cdot10^{-4}$, respectively). Knowing O and H speciation (see Supplementary Note 1), it can be concluded that the exchanged fractions of mobile O (molecular water and OH groups) and mobile H are 5.3 ± 1 % and 6.5 ± 1.3 %, respectively. These two values are close, meaning that the same population of accessible water molecules and OH groups has been exchanged and that the exchanged fraction of mobile O value is similar to that obtain for non-frozen samples.

To conclude, this experiment demonstrates that the precautions taken for the sample preparation in this study are sufficient to obtain reliable O depth profiles and that a cryo-preparation is not required for the analysis of O mobility in this type of materials.

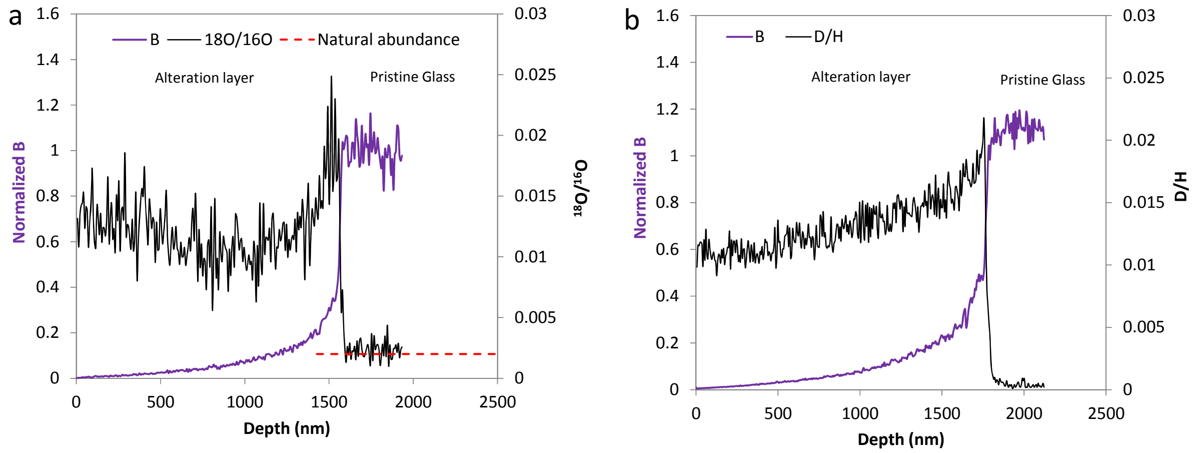

Supplementary Figure 2 – Cryo-ToF-SIMS analysis of the sample ISG-363. **a** B and $^{18}O/^{16}O$ profiles. **b** B and D/H profiles. In both figures, B concentration is normalized to that in the pristine glass. Both isotopic ratios are quantitative, they correspond to the ratio of counts received by the detector for $^{18}O^-$, $^{16}O^-$, D^- and H^-.

ToF-SIMS profiles with opposite sputtering directions

A thin flake of ISG glass was prepared manually by melting ISG glass powder at ~ 1400 °C and blowing a tiny piece of molten glass against a flat graphite bar. This protocol provided several flat flakes of a few square millimeters, each 5 to 10 µm thick (Supplementary Figure 3a). A ~ 5 µm thick flake was selected and altered at 90 °C for 29 hours in a solution containing 160 mg·L^{-1} of Si (saturation with SiO_2am), and 2 g·L^{-1} of K. The $pH_{90\,°C}$ was maintained around 5.5. A ~ 1.4 µm thick alteration layer was formed on both sides of the glass specimen. When removed from the leaching solution, the glass flake was quickly rinsed with deionized water, and then placed for 3 hours at room temperature in $H_2{}^{18}O$. The humid altered flake was then placed on a carbon tape and mounted on the sample holder, then quickly introduced in the vacuum chamber of the SIMS. The external face of the sample remained in contact with air for approximately 3 minutes. Two profiles were recorded: the first for positive secondary ions (analysis beam: 25 keV 2 pA Bi_1^+, abrasion beam 1 keV, 220 nA O_2^+, abraded area 200 µm × 200 µm, analyzed area 50 µm × 50 µm) and the second for negative secondary ions (analysis beam: 25 keV 0.06 pA Bi_3^{++}, abrasion beam 2 keV, 210 nA Cs^+, abraded area 200 µm × 200 µm, analyzed area 50 µm × 50 µm).

Supplementary Figure 3b and 3c show that the B profiles are the same regardless of the direction of sputtering and analysis of the alteration layer, meaning that the B profiles (often displayed and discussed in the literature) are not affected by beam artefacts. However, the enrichment of ^{18}O is not the same in the two layers. A lower value of the $^{18}O/^{16}O$ ratio in layer 1, which was in contact with air for approximately 3 minutes, compared to layer 2 indicates a likely exchange between pore water and air humidity. Nonetheless, the difference of the mean value of $^{18}O/^{16}O$ in the two layers (0.033 vs 0.041) is low relative to the value expected in the case of a complete exchange between pore water and bulk water (0.2). This difference between the O isotopic ratios in the two layers (~ 20 %) provides a good estimation of the uncertainty of the present measurements since they have all been recorded in the same conditions. Furthermore, the increase of ^{18}O enrichment near the reactive interface, already noticed in many ToF-SIMS analyses, is meaningful as it is observed in the two profiles. One can then rule out the hypothesis that water molecules are "pushed" by collisions with ions of the two beams. However, this phenomenon can be seen for K, whose profiles are shifted by approximately 70 nm and the enrichment in the interfacial area in layer 1 corresponds to a depletion in the same area of layer 2.

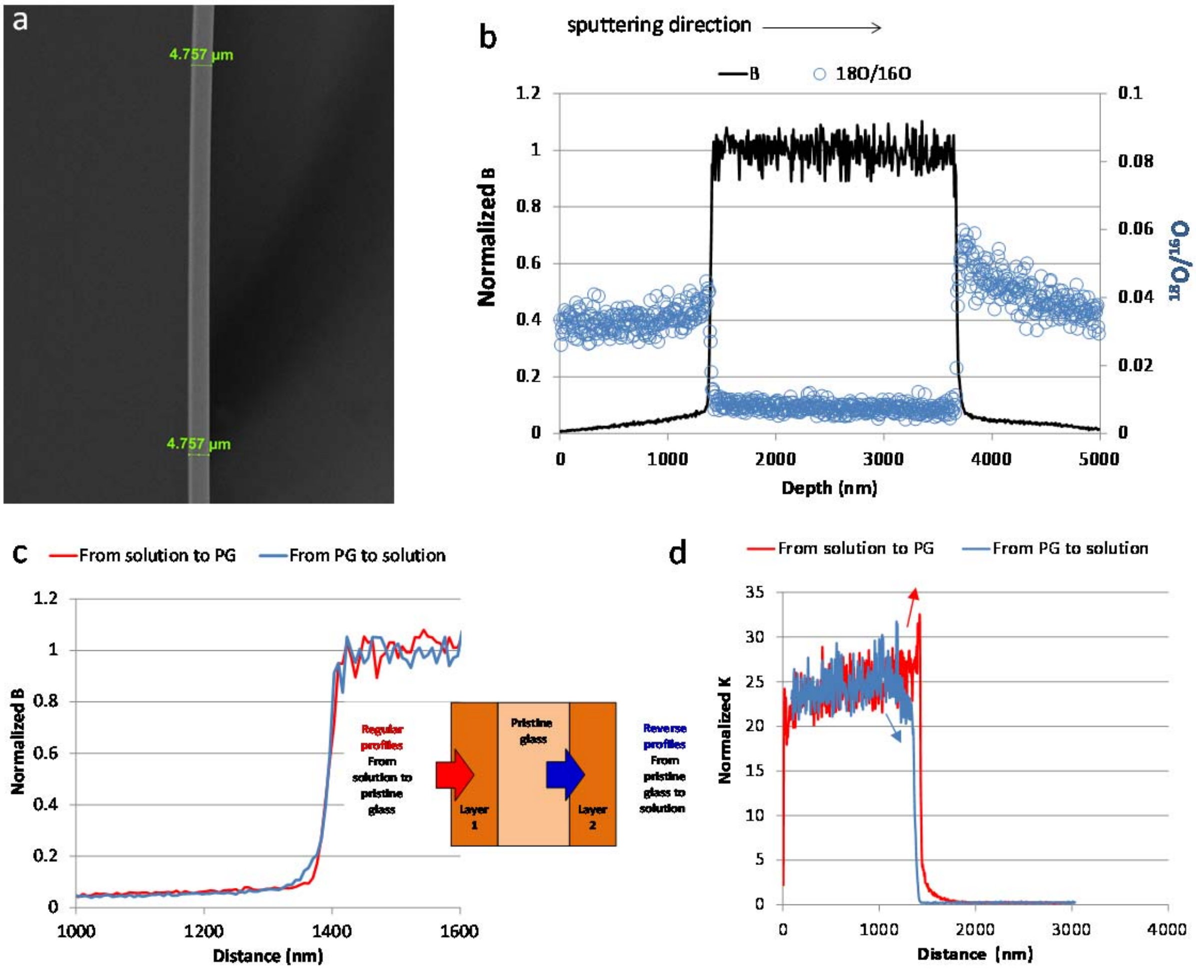

Supplementary Figure 3 – ToF-SIMS analysis of a thin altered film of ISG glass. **a** SEM image of the flake of ISG glass prior to its alteration. **b** ToF-SIMS profiles obtained in negative mode showing the symmetrical B profile delimiting the two alteration layers (layers 1 and 2) and the O isotopic ratio profile that matches natural abundance in the pristine glass but is highly enriched in ^{18}O in the alteration layers. **c,d** Comparison of the B and K profiles in the two layers, recorded in positive mode. To facilitate the interpretation, the blue curves recorded in layer 2 are plotted after transformation upon vertical symmetry and alignment at the depth where C(B) = 0.5. Unlike the B profiles, the K profiles are shifted by 70 nm and the concentration gradients at the reactive interface are opposite (arrows).

Supplementary Note 3 – A model for water diffusion in passivating gel

A model of water diffusion in the gel was developed to rationalize the ToF-SIMS measurements and extract water diffusion coefficients.

Conceptual and mathematical models

The time evolution of the $^{18}O/^{16}O$ profiles (Figure 2) implies the presence of two modes of O exchange within the gel: a first exchange mode that is very rapid but only involves a small fraction of the total O and a second exchange mode that is much slower but for which the amount of exchanged O is not limited within the timescale of the experiments. As illustrated in Supplementary Figure 3, exchange mode 1 is interpreted as resulting from diffusion of isotopically-labeled water molecules in connected pores that run through the gel and link the ^{18}O-enriched aqueous solution to the pristine glass, whereas exchange mode 2 results from diffusion of water out of the connected pores, through the gel, and into closed pores. The latter mechanism is rate limited by the re-organization of the gel, which allows for water molecules in closed pores to be made available for isotopic exchange.

To represent this behavior in a mathematical model, the gel is assumed to contain spherical pores with a single mean pore radius, *r*. Some fraction of the pores are connected in aggregates that run from the solution/gel interface to the gel/pristine glass interface and are thus treated as cylinders with a single mean separation distance, $\langle r_c \rangle$, for simplicity. The connected pores serve as a source for water diffusion into the gel, which allows isotopically-labeled oxygen atoms to reach and exchange with water molecules in closed pores.

Because of the significant difference in timescale between the two exchange modes, they are modeled by two 1D diffusion models that treat "vertical" water diffusion through connected pores with a diffusion coefficient D_c (i.e., normal to the gel surface (z); Section 3.2) and "lateral" water diffusion with a diffusion coefficient D_d (i.e., parallel to the gel surface (x); Section 3.3) separately. In what follows, experimental data and geometric arguments are first used to derive the mean pore radius and mean distance between cylindrical pores and the diffusion models used to treat the two exchange modes and extract water diffusion coefficients are then presented.

Mean pore radius and mean distance between cylindrical pores

From the analysis presented in Supplementary Note 1, we know that 29 % and 71 % of the protons in the gel are from hydroxyls and water molecules, respectively. We also know that 0.26 % of the oxygen atoms in the pores are within the rapidly-exchanging connected pores. Therefore, based on the model presented in Supplementary Figure 3, we can write the following equations from geometric arguments:

$$n_{\mathrm{OH}} = 0.29 = \frac{\rho_{\mathrm{OH}}(A_c n_c/n_t + A_s\, n_s/n_t)}{n_{\mathrm{tot.}}} \tag{11}$$

$$n_{\mathrm{H_2O}} = 0.71 = \frac{2 \times \rho_{\mathrm{H_2O}}(V_c n_c/n_t + V_s\, n_s/n_t)}{n_{\mathrm{tot.}}} \tag{12}$$

$$n_{\mathrm{cyl.}} = 0.0026 = \frac{\rho_{\mathrm{OH}} A_c\, n_c/n_t + \rho_{\mathrm{H_2O}} V_c\, n_c/n_t}{\rho_{\mathrm{OH}}(A_c\, n_c/n_t + A_s\, n_s/n_t) + \rho_{\mathrm{H_2O}}(V_c\, n_c/n_t + V_s\, n_s/n_t)} \tag{13}$$

where:

$$n_{\mathrm{t}} = n_c + n_s \tag{14}$$

$$n_{\text{tot.}} = \rho_{\text{OH}}(A_c\, n_c/n_t + A_s\, n_s/n_t) + 2 \times \rho_{\text{H}_2\text{O}}(V_c\, n_c/n_t + V_s\, n_s/n_t) \tag{15}$$

and n_c and n_s are the number of connected and closed pores, respectively, A_c and V_c are the area and volume of a cylindrical (connected) pore, respectively, and A_s and V_s those of a spherical (closed) pore and are simply defined as:

$$A_{\text{c}} = 2\pi r h \tag{16}$$

$$A_{\text{s}} = 4\pi r^2 \tag{17}$$

$$V_{\text{c}} = \pi r^2 h \tag{18}$$

$$V_{\text{s}} = 4/3\, \pi r^3 \tag{19}$$

where r is the mean pore radius and h is the thickness of the gel. The formula for A_c only considers the pore walls. Solving Eqs. 11-13 for the unknown r yields 0.58 nm.

Considering a cubic geometry, we can calculate a total volume V_t and the corresponding volume of the porosity V_p:

$$V_t = h^3 \tag{20}$$

$$V_p = \rho_p \times V_t$$

where ρ_p is the pore fraction (0.22, see Table S3). We can then calculate the value of n_c and n_s in V_t considering:

$$n_c = \frac{0.0025 \times V_p}{\pi \times r^2 \times h} \tag{21}$$

$$n_s = \frac{0.9975 \times V_p}{\frac{4}{3}\pi \times r^3} \tag{22}$$

To relate <r_c> and <r_s> (the mean distance between two spherical pores) to these quantities, we take a 2D cross section of the gel parallel to the gel/pristine glass interface. For a homogeneous distribution of pores, <r_c> can be defined as:

$$\langle r_c \rangle = \sqrt{\frac{h^2}{n_c}} - 2r \tag{23}$$

$$\langle r_s \rangle = \sqrt{\frac{h^2}{n_s{}^{2/3}}} - 2r \tag{24}$$

The value of <r_c> and <r_s> thus obtained are 43 nm and 0.4 nm, respectively.

Water diffusion in connected pores

Water diffusion in connected pores is modeled simply by using the Crank solution of Fick's second law for a Cartesian dimensional case with boundary conditions $C = C_0$ for $z = 0$ at $t > 0$ and the initial condition $C = 0$ for $z > 0$ and $t = 0$:

$$C(z,t)/C_0 = \text{erfc}\left(z/\left(2\sqrt{D_c t}\right)\right) \tag{25}$$

Supplementary Figure 4 shows a series of calculated profiles with a range of values of D_c and for t = 3 min (the time of the first ToF-SIMS measurement). The profiles show that, for the known gel

thickness of approximately 1500 nm, the water diffusion coefficient in connected pores calculated from MD simulation ($D_c \sim 10^{-10}$ $m^2 \cdot s^{-1}$) is largely sufficient to achieve complete renewal of the water molecules initially in connected pores by the ^{18}O-enriched solution, thus achieving isotopic equilibrium. This simple model does not implement a wall at the gel/pristine glass interface at 1500 nm, but this is not necessary as the current approach is sufficient to demonstrate complete exchange in connected pores within 3 min.

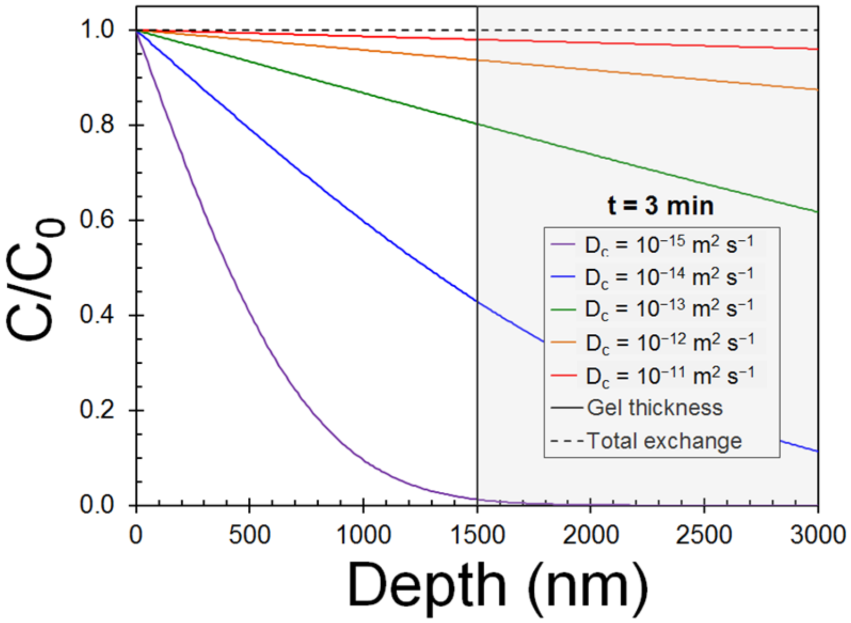

Supplementary Figure 4 – Normalized concentration of water (C/C_0) after 3 min for a range of water diffusion coefficients. The vertical solid line denotes the gel thickness.

Water diffusion through closed pores

Because of the fast diffusion in connected pores, those pores are rapidly in isotopic equilibrium with the ^{18}O-enriched solution and can serve as a continuous source of ^{18}O that diffuses through the gel, via gel re-organization, to reach closed pores. Because the connected pores are represented as cylinders, the water diffusion coefficient in this exchange mode, D_d, is derived from a 1D diffusion model with cylindrical geometry. In this geometry, where the diffusion region is bounded internally by a cylinder with radius a and with a surface maintained at concentration C_0, the Crank solution[3] of Fick's second law becomes:

$$\frac{C(x,t)}{C_0} = 1 + \frac{2}{\pi}\int_0^{\infty} \exp(-D_d u^2 t)\frac{J_0(ur)Y_0(ua) - Y_0(ur)J_0(ua)}{J_0^2(ua) + Y_0^2(ua)}\frac{du}{u} \qquad (26)$$

where J_0 and Y_0 are the Bessel function of order zero of the first and second kind, respectively. Analytical approximations to Eq. 26 for large times are generally complex and involve Laplace transforms. The following simple approximation was derived by Syring and Claassen[4] by limiting the Laplace transform to the first order:

$$\frac{C(x,t)}{C_0} = 1 - \frac{2}{\pi}\arctan\left(\frac{\pi}{ln\frac{4\tau}{\varsigma^2}}\right) ln\frac{x}{a} \qquad (27)$$

where the non-dimensional variable τ is $D_d \times t/a^2$ and $\varsigma = e^{\gamma}$, $\gamma = 0.57721$ being Euler's constant. Eq. 27 is valid for large times, defined by the variable $\tau >> 1$.

The equivalent diffusion surface Σ_{eq} can be obtained by integrating Eq. 27 from $x = a$ to $aW(\tau')$ where $\tau' = 4\tau/\zeta^2 \approx 1.26\tau$ and $W(\tau') = \exp\left(\frac{\pi}{2\arctan\left(\frac{\pi}{\ln(\tau')}\right)}\right)$. The upper bound of the spatial integration corresponds to the value of x for which the linearized approximation of the concentration (Eq. 27) is equal to zero. This gives:

$$\Sigma_{eq}(\tau') = \int_{a}^{aW(\tau')} \frac{C(x',t)}{C_0} 2\pi x' dx' \tag{28}$$

$$\Sigma_{eq}(\tau') = 2\pi a^2 \int_{1}^{W(\tau')} \left(1 - \frac{\ln w'}{\ln\big(W(\tau')\big)}\right) w' dw' \tag{29}$$

$$\Sigma_{eq}(\tau') = \pi a^2 \left(\frac{U(\tau') \quad - 1}{\ln\big(U(\tau') \quad\big)} - 1\right) \tag{30}$$

where $U(\tau') = (W(\tau'))^2 = \exp\left(\frac{\pi}{\arctan\left(\frac{\pi}{\ln(\tau')}\right)}\right)$.

The calculated fraction of the total gel covered, $F_{\mathrm{calc.}}$, at time t is thus:

$$F_{\mathrm{calc.}}(\tau') = \frac{\Sigma_{eq}(\tau')}{\pi\langle r_c\rangle^2} = \left(\frac{a}{\langle r_c\rangle}\right)^2 \left(\frac{U(\tau') \quad - 1}{\ln U(\tau')} - 1\right) \tag{31}$$

The same quantity can also be expressed as a function of the $^{18}O/^{16}O$ ratio in the gel measured from ToF-SIMS and the known isotopic ratio of the solution, $(^{18}O/^{16}O)_{sol}$:

$$F_{\mathrm{exp.}}(t) = \frac{\left(^{18}\mathrm{O}/^{16}\mathrm{O}\right)_t}{\mathrm{O_{ex}}\left(^{18}\mathrm{O}/^{16}\mathrm{O}\right)_{\mathrm{sol}}} \tag{32}$$

where the fraction of exchangeable oxygen O_{ex} exchanged at time t is defined as:

$$\mathrm{O_{ex}} = n_{\mathrm{O_{H_2O}}} + n_{\mathrm{O_{NBO}}} \tag{33}$$

and $n_{\mathrm{O_{H_2O}}}$ and $n_{\mathrm{O_{NBO}}}$ are defined above in Eq. 11 and 12.

The diffusion coefficients required to reproduce the experimentally-derived values of $F_{\mathrm{exp.}}(t)$ can be obtained using Eq. 31 and the corresponding inverse functions:

$$\tau(F_{\text{exp.}}) = \frac{D_d t(F_{\text{exp.}})}{a^2} = \frac{1}{1.26}\exp\left(\pi\cot\frac{\pi}{\ln\left(\left(1+\left(\frac{\langle r_c\rangle}{a}\right)^2 F_{\text{exp.}}\right)G^{-1}\left(\ln\left(1+\left(\frac{\langle r_c\rangle}{a}\right)^2 F_{\text{exp.}}\right)+\frac{1}{1+\left(\frac{\langle r_c\rangle}{a}\right)^2 F_{\text{exp.}}}\right)\right)}\right) \tag{34}$$

where $G^{-1}(A)$ is the inverse function of $G(x) = x - \ln x$ and is computed iteratively using $A + \ln(x)$ starting from $x = A$. For large enough values of the quantity $\left(\frac{\langle r_c\rangle}{a}\right)^2 F_{\text{exp.}}$, Equation (3.15) can be simplified to:

$$\tau(F_{\text{exp.}}) \approx \frac{1}{1.26}\left(\frac{\langle r_c\rangle}{a}\right)^2 F_{\text{exp.}} \ln\left(\left(\frac{\langle r_c\rangle}{a}\right)^2 F_{\text{exp.}}\right) \tag{35}$$

Solving Eq. 34 (or 35) for each experimental data point (from $t = 0$ to the time corresponding to the point) yields values of D_d that decrease with time following a power law (Supplementary Figure 5). D_d decreases to ~ $2{\cdot}10^{-21}$ $m^2{\cdot}s^{-1}$ within 24 h and a value of $6{\cdot}10^{-23}$ $m^2{\cdot}s^{-1}$ is reached for the last experimental data point (2184 h). Values of τ' range from approximately 20 to 1600 and are therefore much greater than 1, as assumed above. The decrease of the diffusion coefficient with time may be indicative of a gel that is becoming less permeable to water diffusion through self-reorganizing and maturing. In addition, the time dependence of the fraction of gel covered, F, varies with geometry; for example, spherical 1D diffusion has a linear dependence on time, Cartesian 1D diffusion is proportional to $\sqrt{t}$ and cylindrical diffusion is approximately proportional to $t/\ln(t)$. Therefore, the observed time dependence of F and D_d may also reflect in part a more complex space dependence than the simple cylindrical geometry adopted in this model.

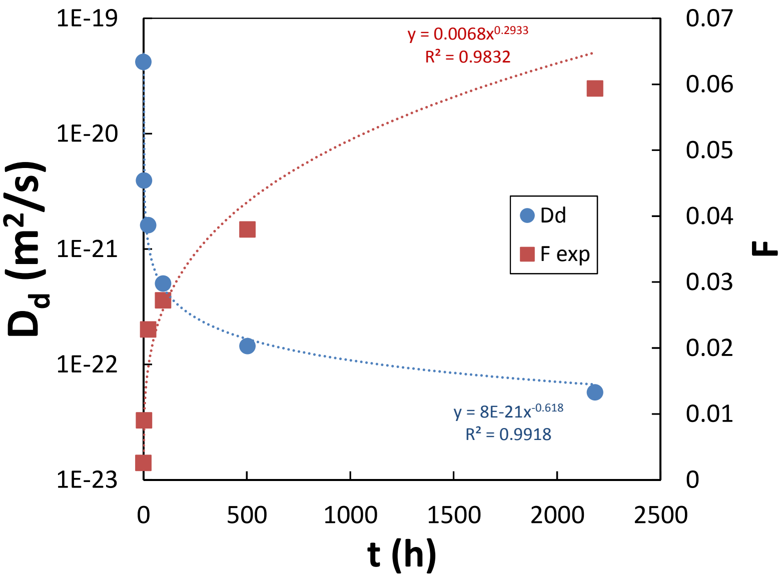

Supplementary Figure 5 – Fraction of gel covered by lateral diffusion of water molecules and corresponding water diffusion coefficient as a function of time.

Water flux at the gel/pristine glass interface

The total flux of water molecules at the gel/pristine glass interface, J_t, is defined as:

$$J_t = J_1 + J_2 \quad (36)$$

where the flux due to exchange mode *i* is:

$$J_i = D_i \times S/V \times \rho_{\mathrm{H_2O}} \times \rho_p \times n_i/n_t \quad (37)$$

where *S*/*V* is the surface-to-gel-volume ratio, which is the reciprocal of the gel thickness, ρ_p is the fraction of pores. The values of J_i thus obtained are $\sim\cdot 10^{-3}$ and $\sim\cdot 10^{-11}$ mol·m^{-2}·s^{-1} for exchange modes 1 and 2 (at long time), respectively, yielding a total flux at the gel/pristine glass interface, J_t, of $\sim\cdot 10^{-3}$ mol·m^{-2}·s^{-1} controlled by the transport through connected pores. This flux can be compared to that estimated in a non-passivating gel of the same thickness ($D \sim 10^{-10}$ m^2·s^{-1}, $\rho_p \sim 0.9$, $n_i/n_t \sim 0.9$), $J_{max} \sim 30$ mol·m^{-2}·s^{-1}. The difference of approximately 4 orders of magnitude is mostly explained by the small fraction of connected pores in the passivating gel.

Supplementary Note 4 – Molecular dynamics simulations

MD simulation of water diffusion in cylinder pores

Water diffusion in silica micropores[5,6] was studied by molecular dynamics (MD) with reactive dissociable water potential (MGFF)[7,8]. These MGFF potentials have been used to study reactivity of water on glass surfaces and the structure and dynamics of water under confinement. Simulations were performed using the LAMMPS code[9]. Visualization and some analyses were performed with VMD code[10]. To construct the silica-pore structures, cubic blocks of cristobalite silica 4 nm × 4 nm × 6 nm (6 nm × 6 nm × 6 nm for the 4 nm pore) were generated and atoms were removed to create pore with radii of 0.5 nm, 1 nm, 2 nm, or 4 nm. Care was taken to maintain the SiO_2 stoichiometry upon removal of Si and O atoms by adjusting the radius by up to 0.1 Å. The crystalline structure with the cylindrical pore was melted at 5298 K and gradually cooled to 298 K under NVT conditions. Constraints of reflecting walls and reflective cylinder at the center of the pore were placed to prevent atoms from occupying these regions. The melt quenched structure was then annealed to 1298 K and cooled back to 298 K with the reflecting regions. Blocks of water about 3 nm thick were added to both sides of the structure as well as inside the pore at a density of approximately 33 molecules per nm^3 corresponding to a density of ~ 1 $g{\cdot}cm^{-3}$. A schematic depiction of the setup is shown in Figure 4 in the article with a portion of the silica structure sectioned off to show water inside the pore. NVT simulations at 298 K were performed for all the systems for 8 ns for the 0.5 nm and 1 nm pore systems, 4 ns for the 2 nm pore system and 2 ns for the 4 nm pore system. Longer time of simulations for smaller pores was due to their lower water diffusion coefficients. Diffusion coefficients of each water molecule was calculated based on their displacement over 10 ps and this value of the diffusion coefficient was mapped onto the initial position of the oxygen to obtain a 3D diffusion map. The diffusion coefficients shown in Figure 4 in the article were calculated as the average of diffusion coefficient in the radial direction over the length of the pore. Diffusion coefficients were also calculated for the 1 nm pore system with the ReaxFF potential. We observed that the diffusion coefficient was reduced at the center of the pore albeit to a lesser extent than with the MGFF potentials. The Hole program[11] was used to determine the location of the pore walls and is shown in the figure as the vertical dashed lines.

MD simulations of ISG structure and bottleneck, interstial size analyses

By using a set of recently developed composition dependent partial charge potentials that are capable to describe the boron coordination change with composition, glass structures with the ISG glass (composition: 60.20 SiO_2-3.84 Al_2O_3-15.97 B_2O_3-12.65 Na_2O-5.73 CaO-1.62 ZrO_2, in mol%; glass density: 2.52 $g{\cdot}cm^{-3}$) were simulated using the simulated melt and quench process with MD simulations. A combination of constant volume simulations (NVT) during cooling with a cooling rate of 5 $K{\cdot}ps^{-1}$ and relaxation under constant pressure (NPT) at ambient pressure at 300 K were used[12,13]. Detailed of the empirical potentials and glass simulation procedures can be found in reference[12]. A large system size of 24,000 atoms was used to ensure capturing the short and medium range structures of these boroaluminosilicate glasses. Three independent structures were generated with different initial random configuration.

Cation coordination numbers in the ISG system are 4.00 for Si, 4.00 for Al, 3.59 for B, 5.68 for Zr, 7.42 for Na and 6.62 for Ca[12]. These are in good agreement with experimental data for related boroaluminosilicate glasses. For boron coordination, which changes with glass composition (and potentially thermal history), the percentage of 4-fold coordinated boron is 59 % which is in excellent agreement with NMR studies of 52 %[12].

Snapshot of the bulk ISG structure is shown in Supplementary Figure 6. After the generation of the ISG structures, the gel structures were prepared. The first type of gel structure was obtained by removing Na atoms only ("Na-removed"). The second type was prepared by removing all Na, B, 30 % of Ca and the free oxygen ions, i.e. oxygen ions that are not bonded to any other atom within a distance of 2.5 Å.

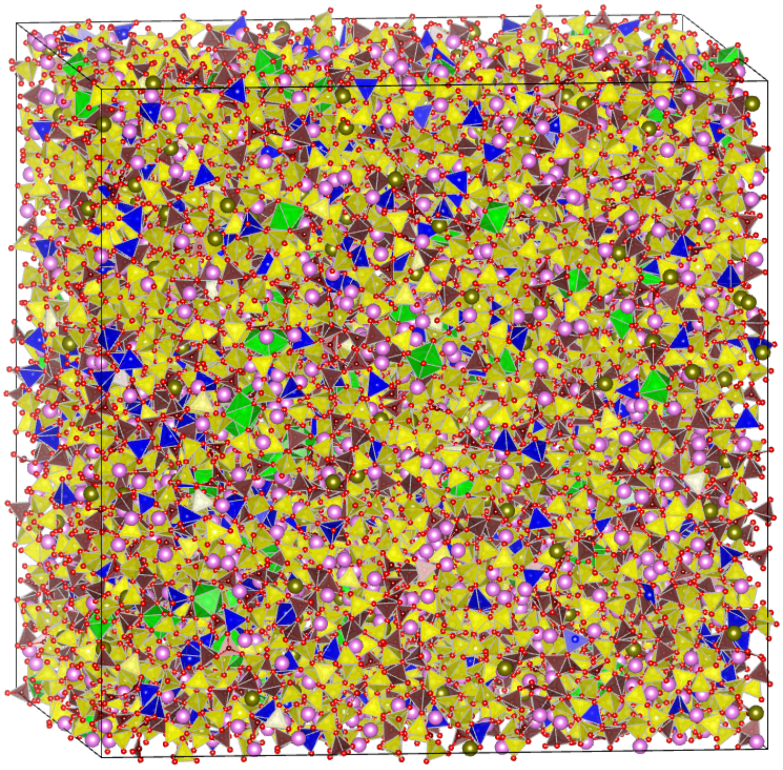

Supplementary Figure 6 – Snapshot of the simulated ISG glass structure. Green polyhedron: ZrO_6, blue tetrahedron: AlO_4, yellow tetrahedron: SiO_4, brown triangle and tetrahedron: BO_3 or BO_4, pink ball: Na, golden ball: Ca. Total number of atoms: 24,000. Simulation cell: 6.7 x 6.7 x 6.7 nm^3.

Interstitial size and bottleneck size analysis

Interstitial (or void) sizes available in the glass structure determine the free volume that water molecules can reside in and bottleneck size determines the available pathways for water diffusion. Both interstitial and bottleneck size distribution were calculated in the bulk, Na leached and gel structures generated from MD simulations. Voronoi-Delaunay tetrahedron analysis was performed using the algorithm developed by Malavasi *et al.*[14] and Mansas *et al.*[15]. Supplementary Figure 7 shows a Delaunay tetrahedron with interstitial site in the middle and a bottleneck site on the face of the tetrahedron.

To calculate the interstitial site and bottleneck distributions, we need to use atomic radii for each of the elements. The atomic radii used were 1.10, 0.41, 0.27, 0.50, 0.95, 0.99 and 0.72 Å for O, Si, B, Al, Na, Ca and Zr, respectively. These values were chosen from atomic radii with a constraint that the sum of radii of two neighboring atoms remain lower than the distance between them[15]. The distribution of interstitial site density distribution and bottleneck site distribution are shown in Fig. 5 (a) and (b). The zoomed picture is shown from 1.28 Å in the paper. As the size of water molecule is around 2.7 Å, any radius larger than 1.28 Å will be able to contain one or more water molecule for interstitial site and will allow water molecule to pass for bottleneck site. These analysis help to understand the gel structure in terms of water solubility and diffusion pathways.

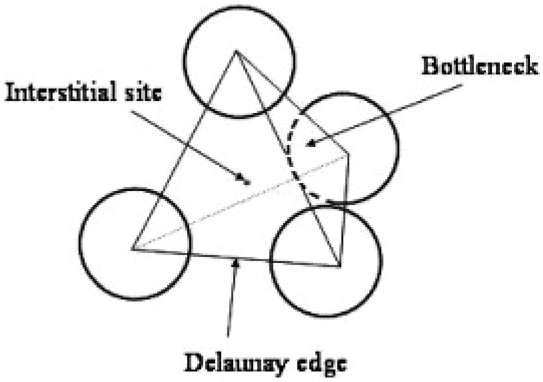

Supplementary Figure 7 – Delaunay tetrahedron in a close packing sphere showing the interstitial site in the center and bottleneck in face center*.

In addition to the analysis of bulk ISG structures, we also performed interstitial and bottleneck site density analyses for Na removed and gel structures[14]. For Na-removed structure, all Na atoms were removed considering the initial stage of ion exchange. For the gel structure, we removed all Na, B and 30 % of Ca. This is due to experimental investigations showing that the 70 % Ca plus Si, Al, and Zr remained in the gel[12]. Ca ions play the role of charge compensators in the gel with all the Na ions leached out. In these analysis, after removing these cations, some oxygen ions that do not bond to any other cation (free oxygen atoms) were removed as well. Supplementary Figure 8 shows the interstitial site (a) and bottleneck site density (b) analysis results in the three types of structures: bulk ISG, Na-removed, and gel structure.

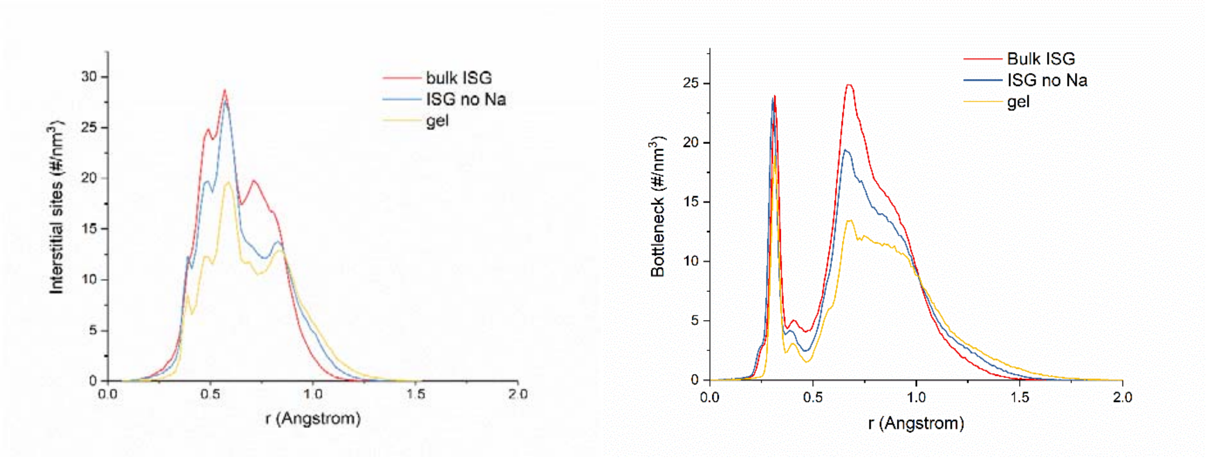

Supplementary Figure 8 – Interstitial and bottleneck analysis. a Interstitial site size distribution. **b** Bottleneck size distribution for ISG bulk, Na-removed ISG and ISG gel structure.

* Reprinted from *Journal of Non-Crystalline Solids*, volume 352, issue 3, Gianluca Malavasi, M. Cristina Menziani, Alfonso Pedone, Ulderico Segre, Void size distribution in MD-modelled silica glass structures, pages 285-296, Copyright (2006), with permission from Elsevier.

Supplementary References

1 Zhuravlev, L. T. The surface chemistry of amorphous silica. Zhuravlev model. *Colloids Surf. A: Physicochem. Engin. Asp*. **173**, 1-38 (2000).

2 Ide, M. *et al*. Quantification of silanol sites for the most common mesoporous ordered silicas and organosilicas: total versus accessible silanols. *Phys. Chem. Chem. Phys.* **15**, 642-650 (2013).

3 Crank, J. *The Mathematics of Diffusion*, Oxford University Press, 2nd Ed., p. 87, Eq. 5.77 (1975).

4 Syring, K.M. & Claassen, N. Estimation of the influx and the radius of the depletion zone developing around a root during nutrient uptake. *Plant and Soil* **175**, 115-123 (1995).

5 Zhuravlev, L. T. The surface chemistry of amorphous silica. Zhuravlev model. *Colloids Surf. A: Physicochem. Engin. Asp.* **173**, 1-38 (2000).

6 Ide, M. *et al.* Quantification of silanol sites for the most common mesoporous ordered silicas and organosilicas: total versus accessible silanols. *Phys. Chem. Chem. Phys.* **15**, 642-650 (2013).

7 Mahadevan, T. S. & Garofalini, S. Dissociative water potential for molecular dynamics simulations. *J. Phys. Chem. B* **111**, 8919-8927 (2007).

8 Mahadevan, T. S. & Garofalini, S. Dissociative chemisorption of water onto silica surfaces and formation of hydronium ions. *J. Phys. Chem. C* **112**, 1507-1515 (2008).

9 Plimpton, S. *LAMMPS website*, <http://lammps.sandia.gov/> (2003).

10 Humphrey, W., Dalke, A. & Schulten, K. VMD - Visual Molecular Dynamics, *J. Molec. Graphics* **14**, 33-38 (1996).

11 Smart, O. S., Goodfellow, J. M. & Wallace B. A. The Pore Dimensions of Gramicidin A. *Biophys. J.* **65**, 2455-2460 (1993).

12 Collin, M. *et al*. Structure of International Simple Glass and properties of passivating layer formed in circumentural pH conditions, *npj Mater. Degrad.* **2**, 4 (2018).

13 Ren M., Deng L. & Du J. Bulk, surface structures and properties of sodium borosilicate and boroaluminosilicate nuclear waste glasses from molecular dynamics simulations, *J. Non-Cryst. Solids* **476**, 162-169 (2017).

14 Malavasi, G., Menziani, M. C., Pedone, A. & Segre, U. Void size distribution in MD-modelled silica glass structures, *J. Non-Cryst. Solids* **352**, 285-296 (2006).

15 Mansas, C. *et al*. Drivers of water transport in glass: chemical or topological effect of the glass network? *J. Phys. Chem. C* **121**, 16201-16215 (2017).
